# Reduced Apparent Diffusion Coefficient in Various Brain Areas following Low-Intensity Transcranial Ultrasound Stimulation

**DOI:** 10.3389/fnins.2017.00562

**Published:** 2017-10-09

**Authors:** Yi Yuan, Yanchao Dong, Shuo Hu, Tao Zheng, Dan Du, Juan Du, Lanxiang Liu

**Affiliations:** ^1^Institute of Electrical Engineering, Yanshan University, Qinhuangdao, China; ^2^Department of Magnetic Resonance Imaging, Qinhuangdao Municipal No. 1 Hospital, Qinhuangdao, China

**Keywords:** focused ultrasound stimulation, diffusion-weighted MRI, apparent diffusion coefficient, rat, brain

## Abstract

Diffusion of water molecules closely related to physiological and pathological information of brain tissue. Low-intensity transcranial ultrasound stimulation (TUS) has advantages of noninvasive, high spatial resolution and penetration depth. Previous studies have demonstrate that TUS can modulate neuronal activity and alter cortical hemodynamic. However, how TUS affect diffusion of water molecules remain unclear. In this paper, in order to evaluate the effect of low-intensity TUS on the diffusion of water molecules in brain tissue, diffusion-weighted magnetic resonance (MR) imaging was performed in 19 healthy Sprague-Dawley rats in sham surgery group (six rats) and TUS group (thirteen rats) Subsequently, rats were stimulated by low-intensity transcranial ultrasound for 5 min in TUS group. Finally, rats of sham surgery group and TUS group were imaged again by diffusion-weighted MR imaging. The apparent diffusion coefficient (ADC) was measured in caudate putamen region and middle brain motor-related region of each rat in sham surgery group and TUS group. Surgery-related and TUS-related changes were calculated using a statistical analysis. The mean ADC values of marked regions of six rats in sham surgery group were 0.743 ± 0.031 (pre-surgery) and 0.745 ± 0.029 (post-surgery). The mean ADC values of marked regions of 13 rats in TUS group were 0.749 ± 0.032 (pre-TUS) and 0.712 ± 0.033 (post-TUS) Compared to the pre-TUS values, the mean ADC values of the rats decreased 4.9% (^*^P < 0.05) post-TUS. These results of this study demonstrate that low-intensity TUS can restrict the diffusion of water molecules in brain tissue.

## Introduction

Diffusion-weighted magnetic resonance (MR) imaging, which is based on the random and irregular Brownian motion of water molecules, reflects the different states of water molecules in tissue (Engelbrecht et al., 2002; Fernández-Espejo et al., 2011). It can effectively reflect the internal microstructure and micromotion and can provide the functional status of human tissues at the molecular level (Malayeri et al., 2011; Chavhan et al., 2014). It is the only available method for the noninvasive detection of living tissue water molecule diffusion, has high sensitivity and can provide very valuable information for diagnosing of lesions (Pienaar et al., 2012; Koutoulidis et al., 2017). Diffusion-weighted MR imaging also plays an important role in the diagnosis of brain diseases such as acute cerebral ischemia, brain tumors, stages of brain abscess, and diffuse axonal injury. It is of great significance that apparent diffusion coefficient (ADC) values can be used for the quantitative assessment of the aforementioned diseases (Schaefer et al., 2000).

Compared with transcranial magnetic stimulation and transcranial direct current stimulation, both noninvasive brain stimulation technologies commonly used for neuromodulation in the clinic, noninvasive transcranial ultrasound stimulation (TUS) has high spatial resolution and deep penetration (Bystritsky et al., 2011; Bystritsky and Korb, 2015; Naor et al., 2016). The mechanism of TUS is based on an ultrasound-induced complex mechanoelectrical interplay that leads to excitation, primarily through the effect of currents induced by membrane capacitance changes in neurons (Plaksin et al., 2014). In 1929, Edmund Newton Harvey first demonstrated that ultrasound could stimulate nerve and muscle fibers in neuromuscular preparations (Harvey, 1929). In recent years, more and more groups have focused on TUS for brain modulation and obtained good results. In brain neural activity modulation, low-intensity TUS can stimulate neuronal activity and synchronous oscillations in the intact hippocampus (Tufail et al., 2010, 2011), modulate phase-amplitude coupling of neuronal oscillations from the rat hippocampus (Yuan et al., 2016a,b) and modulate the activity of the primary somatosensory cortex in humans (Legon et al., 2014). Low-intensity TUS can also protect against aluminum-induced cerebral damage in an Alzheimer's disease rat model and suppress chemically induced acute epileptic EEG activity (Min et al., 2011; Lin et al., 2015). In cerebral oxygen saturation, low-intensity TUS was combined with fMRI to monitor the modulation of region-specific brain activity. The results showed that the TUS could alter the BOLD signal (Yoo et al., 2011). In cerebral blood flow, low-intensity TUS was able to increase cerebral blood flow and protect the brain from ischemic injury (Guo et al., 2015). These previous studies have demonstrated that TUS can modulate brain neural activity, cerebral oxygen saturation and cerebral blood flow. However, the effect of TUS on the diffusion of water molecules in brain tissue remains unknown. To explore the phenomenon, this study used diffusion-weighted MR imaging to study the change in ADC in sham surgery group and TUS group with Sprague-Dawley rats and evaluate the effect of TUS on diffusion of water molecules in brain tissue.

Previous investigations have indicated that low-intensity TUS can excite neuron excitability in motor cortex to induce motor responses of rat or mouse limbs and tails. We planned to analyze the changes of ADC in the brain nuclei regions related to motor in our study. However, since the cortex was thin (about 2-3 mm) and closed to the skull, the axial image was easily affected by the skull and cerebrospinal fluid in MRI with slice thickness of 2.0 mm. The measurement of ADC in motor cortex was inaccurate. Therefore, the deep brain nuclei associated with motor responses (caudate putamen region and middle brain motor-related region) was chosen to analyze the change of ADC with TUS.

## Materials and methods

### Animals

A total of 19 Sprague-Dawley rats (male; age, 3-months-old; weight, 250–290 g; Beijing Vital River Laboratory Animal Technology Co., Ltd. Beijing, China) were used for our study (thirteen rats in TUS group, six rats in sham surgery group). Each cage housed two rats at 20–25°C with a 12 h light/dark cycle. All procedures were performed in accordance with the Animal Ethics and Administrative Council of Yanshan University. This study protocol was also approved by Animal Ethics and Administrative Council of Yanshan University. We accorded to “The Tab of Animal Experimental Ethical Inspection, Yanshan University (ID Number: 20160021)” to perform the experiment. Surgical anesthesia was induced with sodium pentobarbital (3%, 5 mg/100 g, IP). The anesthetized rats were fixed on a stereotaxic apparatus (ST-5ND-C, Stoelting Co., USA) with ear bars and a clamping device. The fur of the rat head was shaved, and the skin was cleaned with physiological 0.9% sodium chloride solution. The skin was cut along the midline of the skull, and the subcutaneous tissue and periosteum were cleaned.

### Experimental setup and parameters of TUS

A commercial ultrasonic brain stimulator (DK-102T, Dukang Medical Devices, CO., LTD, Shijiazhuang, China) was used for TUS in the experiment. The fundamental frequency of ultrasound was 800 kHz. The area of the unfocused ultrasound transducer was 3.5 cm^2^. The ultrasound duration and the inter-stimulus interval were 10 and 100 ms, respectively. The spatial-peak and pulse-average intensity (I_sppa_) was ~1.2 W/cm^2^ (Kim et al., 2014). The corresponding spatial-peak temporal-average intensity (I_spta_) was calculated as previously reported and found to be ~0.12 W/cm^2^. The potential temperature increases due to ultrasound parameters can be estimated by the equation (Collins et al., 2004; Lee et al., 2015)^Δ*T* = 2α*It*/*ρC*_*p*_^ using the absorption coefficient α = 0.0175 cm^−1^, ultrasound intensity I = 1.2 W/cm^2^, a TUS duration *t* = 0.001 s, where ρ is the density of brain tissue, Cp is the specific heat of the brain tissue, and ρCp is 3.796 J^*^cm^3^*^°^C^−1^. Therefore, the maximum temperature enhancement induced by TUS would be 1.1^*^10^−4°^C, which is far below the temperature threshold predicted to induce tangible thermal bio-effects.

### Experimental process

TUS group: (1) First, the anesthetized surgery rat was imaged by diffusion-weighted MR imaging in the pre-TUS condition. Subsequently, the rat was fixed on the stereotaxic apparatus, and the skull was smeared with ultrasound coupling gel. (2) The ultrasound transducer was placed on the surface of the ultrasound coupling gel, and the distance between the skull and the transducer was 2 mm. The rat was stimulated by low-intensity transcranial ultrasound for 5 min. (3) Finally, the rat was imaged by diffusion-weighted MR imaging again to obtain the post-TUS data.

Sham surgery group: In the sham surgery the experimental process was the same to (1) and (3) in the TUS group. The photography of the surgery rats was shown in Supplementary Figure 1.

### MR image acquisition and post-processing

**Figure 1:**
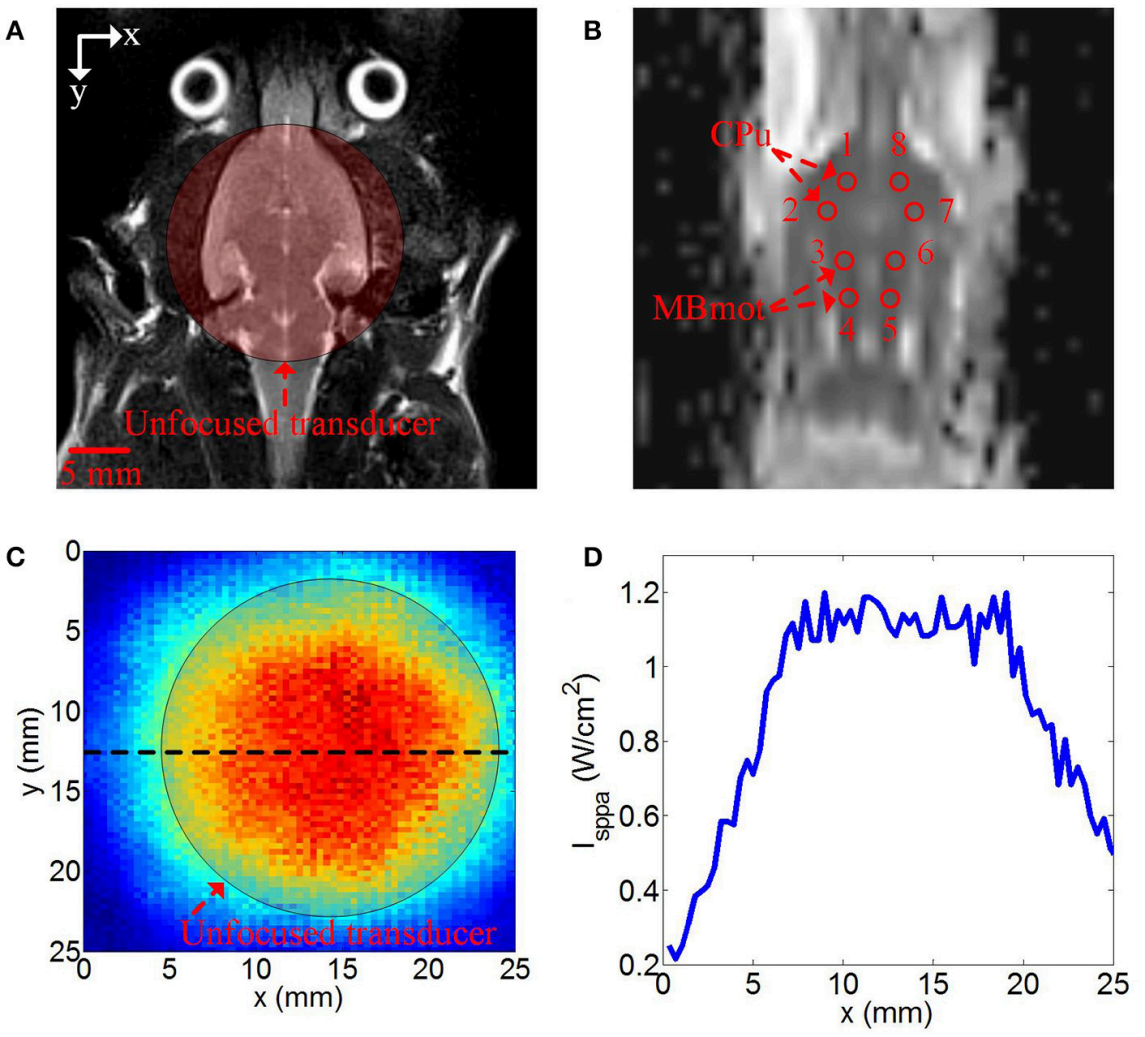
**(A)** Axial T2 image of rat and the position of unfocused transducer on the surface of the rat skull, **(B)** DWI image of rat, the red circle was the regions of interest (ROIs) for calculating ADC values, ROI1 and ROI2 were caudate putamen (CPu) region, ROI3 and ROI4 were middle brain motor-related (MBmot) region. **(C)** two-dimensional ultrasound distribution in x-y plane, **(D)** reconstructed profile of ultrasound distribution marked by the black dotted line in **(C)**.

The MR parameters were as follows: axial T2WI (Figure 1A) turbo spin-echo (TSE) (repetition time [TR]/echo time [TE] 3,000/113 ms, flip angle = 150°, field of view [FOV] 74 × 74 mm, average = 14, voxel size = 0.3 × 0.3 × 2.0 mm, slice thickness = 2.0 mm, number of slices = 10); transverse T2-weighted TSE (TR/TE 4,000/111 ms, flip angle = 150°, average = 10, FOV = 70 × 70 mm, voxel size = 0.4 × 0.3 × 1.0 mm, slice thickness = 2.0 mm, number of slices = 12); Single-shot spin echo- echo echo-planar imaging (SE-EPI) was used in DWI. Scan layers were aligned parallel to the anterior/posterior line with the following settings: TR/TE 3,200/95 ms, FOV = 65 × 65 mm, slice thickness thickness = 1.8 mm, voxel size = 1.5 × 1.0 × 1.8 mm, number of slices = 10, b-value parameter of 0 and 1,000 s/mm^2^ respectively. To increase the signal-to-noise ratio, scanning was repeated nine times (total scanning time = 2 min 13 s).

Imaging analysis was carried out using prototype software on a workstation (Siemens Verio 3.0T MR Leonardo 3682). The ADC parameter image maps of rat brains were co-registered with T2WI rat brain images for exact identification of the striatum and brainstem. A radiologist with 10 years of experience in neural MR imaging, who was blinded to the animal grouping, placed eight circular regions of interest (ROI) measuring 0.5 cm^2^ from anterior striatum to brainstem on both sides (Figure 1B).

### Ultrasound distribution

The position of unfocused transducer on the surface of the rat skull was shown in Figure 1A. The two-dimensional ultrasound distribution was measured by a calibrated needle-type hydrophone (HNR500, Onda, Sunnyvale, CA) that was moved by two dimensional electric translation platform. The two-dimensional ultrasound distribution in x-y plane was shown in Figures 1C,D was the reconstructed profile of ultrasound distribution marked by the black dotted line in Figure 1C. The ultrasound distribution was approximately uniform in the stimulation region.

### Calculation of mean ADC values

In our study, CPu regions and MBmotregions were chosen to calsulate the mean ADC values. As shown in Figure 1B, the ROI 1 and ROI 2 were CPu regions, ROI 3 and ROI 4 were MBmot regions in the right hemisphere of rat. The ROI 5, ROI 6, ROI 7, and ROI 8 in the left hemisphere of rat were the symmetrical regions to ROI 4, 3, 2, 1. The mean ADC values were evaluated quantitatively with signal intensity measurements. With ROI analysis, we measured mean ADC values of each ROI of all rats in sham surgery group and TUS group.

### Statistical analysis

Statistical analysis was conducted using SPSS statistical software, version 21.0 (IBM SPSS Statistics for Windows, Armonk, New York, USA). The ADC values were represented as the mean ± standard deviation (S.D). The significance of the difference between pre-surgery and post-surgery, or pre-TUS and post-TUS was evaluated with a Friedman test, and differences were considered significant when *P* < 0.05.

## Results

**Figure 2:**
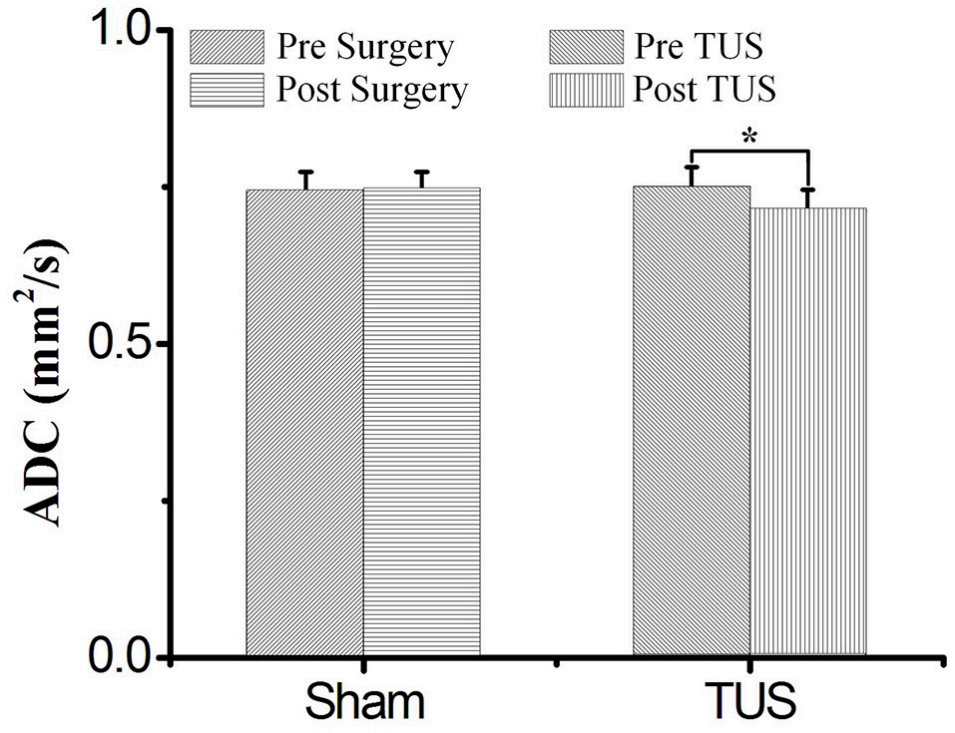
The total mean ADC values in sham group and TUS group. In the sham group, the mean ADC values were 0.743 ± 0.031 (pre-surgery) and 0.745 ± 0.029 (and post-surgery), (*n* = 6, mean ± s.d). In the TUS group, the mean ADC values were 0.749 ± 0.032 (pre-TUS) and 0.7120 ± 0.033 (and post-TUS), (*n* = 13, mean ± s.d, Friedman test, ^*^P < 0.05).

The mean ADC values of TUS group were shown in Table 1. The mean ADC values of all ROIs post-TUS were lower than the values pre-TUS (*n* = 13). There were statistically significant differences in the results from 6 ROIs (ROI 1, ROI 2, ROI 4, ROI 6, ROI 7, and ROI 8, Friedman test, ^*^P < 0.05, *n* = 13). The mean ADC values of sham surgery group were shown in Supplementary Table 1. A statistical analysis of the experimental results for all ROIs of 13 examined rats in TUS group and 6 examined rats in sham surgery group was presented in Figure 2. In sham surgery group, we calculated the total mean ADC values of six rats pre- and post-surgery. The values were 0.743 ± 0.031 (pre-surgery, mean ± s.d, *n* = 6) and 0.745 ± 0.029 (and post-surgery, mean ± s.d, *n* = 6). These results indicated that the surgery did not induce the change of mean ADC values. In TUS group, we calculated the total mean ADC values of 13 rats pre- and post-TUS. The values were 0.749 ± 0.032 (pre-TUS, mean ± s.d, *n* = 6) and 0.712 ± 0.033 (post-TUS, mean ± s.d, *n* = 6). Differences between the values (mean ± s.d) were tested by conducting a Friedman test, with a significance threshold of ^*^P < 0.05). Compared to the pre-TUS results, the mean ADC values decreased 4.9% post-TUS. Thus, the results showed that TUS decreased mean ADC values in the rat cerebral cortex.

**Table 1:** (TUS group) The mean ADC values pre- and post-TUS in different ROIs.

	**Mean ADC ±*SD* (mm^2^/s) (*n* = 13)**		
**ROI**	**pre-TUS**	**post-TUS**	***P*-value**
1	0.765 ± 0.049	0.724 ± 0.025	0.0023^*^
2	0.744 ± 0.060	0.721 ± 0.036	0.0165^*^
3	0.766 ± 0.057	0.740 ± 0.030	0.1715
4	0.806 ± 0.064	0.759 ± 0.051	0.0422^*^
5	0.738 ± 0.041	0.712 ± 0.037	0.2125
6	0.755 ± 0.079	0.709 ± 0.070	0.0017^*^
7	0.704 ± 0.052	0.674 ± 0.062	0.0405^*^
8	0.717 ± 0.047	0.659 ± 0.027	0.0126^*^
ROI 1,2,7,8: CPu	ROI 3,4,5,6: MBmot		^*^P < 0.05

## Discussion

In this study, we qualitatively analyzed the mean ADC values of each rat in pre- and post-TUS conditions, and we observed that TUS decreased the mean ADC values in 11 rats. We also found that the mean ADC values of 13 rats were decreased by TUS, and the result has been expected statistical significance. The results demonstrated that TUS could alter the diffusion of water molecules. However, the mechanism of TUS modulation of ADC values is not yet clear. As we know, the diffusion of water molecules *in vivo* includes extracellular, intracellular and intercellular movements as well as microcirculation. Extracellular movement and microcirculation are the main causes of attenuation of the diffusion-weighted MR imaging signal. Owing to the random motion of water molecules in tissue, the signal attenuation is more obvious in diffusion-weighted MR imaging, and the corresponding ADC values are lower. In this study, we found that the mean ADC values of rat brain tissue were decreased by TUS. The result indicated that the random motion of water molecules decreased under the action of ultrasound. The possible reason for this interesting phenomenon is that, as we know, ultrasound can cause neuronal excitability (Tufail et al., 2010). During excitation, the swelling of astrocytes is accompanied by a reduction in the extracellular space volume of up to 30% (Shi et al., 2015). The reduction of extracellular space volume will decrease the area of molecular water diffusion. Therefore, the neuron activity and the limitation of molecular water diffusion are relevant. The above hypothesis needs further experimental verification. In our future work, we plan to elucidate the mechanism of TUS for altering water molecule diffusion.

Some brain diseases such as acute cerebral ischemia, brain tumors, stages of brain abscess, and diffuse axonal injury can be quantitatively assessed by ADC values. In our study, we found that TUS could decrease ADC values and alter the diffusion of water molecules. In conclusion, in the current study, we demonstrated that TUS can decrease mean ADC values and alter water molecule diffusion in rat brain tissue. This finding has important implications for our understating of the effect of TUS on neuromodulation from the perspective of water molecule diffusion.

## Ethics statement

Animal Ethics and Administrative Council of Yanshan University has approved this study. The animal owner agreed to use these rats in the experiment. The vulnerable populations were not involved in our experiment.

## Author contributions

YY, YD, and LL designed and coordinated the study, YY, SH, TZ, DD, JD, and LL carried out experiment and data process, and drafted the manuscript. All authors gave final approval for publication.

### Conflict of interest statement

The authors declare that the research was conducted in the absence of any commercial or financial relationships that could be construed as a potential conflict of interest.

## References

[B1] Bystritsky A. Korb A. S. (2015). A review of low-intensity transcranial focused ultrasound for clinical applications. Curr. Behav. Neurosci. Rep. 2, 60–66. 10.1007/s40473-015-0039-0

[B2] Bystritsky A. Korb A. S. Douglas P. K. Cohen M. S. Melega W. P. Mulgaonkar A. P.. (2011). A review of low-intensity focused ultrasound pulsation. Brain Stimul. 4, 125–136. 10.1016/j.brs.2011.03.007 21777872

[B3] Chavhan G. B. AlSabban Z. Babyn P. S. (2014). Diffusion-weighted imaging in pediatric body MR imaging: principles, technique, and emerging applications. Radiographics 34, 73–88. 10.1148/rg.343135047 24819803

[B4] Collins C. M. Smith M. B. Turner R. (2004). Model of local temperature changes in brain upon functional activation. J. Appl. Physiol. 97, 2051–2055. 10.1152/japplphysiol.00626.2004 15322067

[B5] Engelbrecht V. Scherer A. Rassek M. Witsack H. J. Mödder U. (2002). Diffusion-weighted MR imaging in the brain in children: findings in the normal brain and in the brain with white matter diseases. Radiology 222, 410–418. 10.1148/radiol.2222010492 11818607

[B6] Fernández-Espejo D. Bekinschtein T. Monti M. M. Pickard J. D. Junque C. Coleman M. R.. (2011). Diffusion weighted imaging distinguishes the vegetative state from the minimally conscious state. Neuroimage 54, 103–112. 10.1016/j.neuroimage.2010.08.035 20728553

[B7] Guo T. F. Li H. D. Lv Y. F. Lu H. Y. Niu J. H. Sun J. F. (2015). Neuroprotective pulsed transcranial ultrasound stimulation in ischemic brain injury after distal middle cerebral artery occlusion. IEEE Trans. Biomed. Eng. 62, 2352–2357. 10.1109/TBME.2015.2427339 25935023

[B8] Harvey E. N. (1929). The effect of high frequency sound waves on heart muscle and other irritable tissues. Am. J. Physiol. 91, 284–290.

[B9] Kim H. Chiu A. Lee S. D. Fischer K. Yoo S. (2014). Focused ultrasound-mediated non-invasive brain stimulation: examination of sonication parameters. Brain Stimul. 7, 748–756. 10.1016/j.brs.2014.06.011 25088462 PMC4167941

[B10] Koutoulidis V. Fontara S. Terpos E. Zagouri F. Matsaridis D. Christoulas D.. (2017). Quantitative diffusion-weighted imaging of the bone marrow: an adjunct tool for the diagnosis of a diffuse MR imaging pattern in patients with multiple myeloma. Radiology 282, 484–493. 10.1148/radiol.2016160363 27610934

[B11] Lee W. Kim H. Jung Y. J. Song I. U. Chung Y. A. Yoo S. S. (2015). Image-guided transcranial focused ultrasound stimulates human primary somatosensory cortex. Sci. Rep. 5:8743. 10.1038/srep08743 25735418 PMC4348665

[B12] Legon W. Sato T. F. Opitz A. Mueller J. Barbour A. Williams A.. (2014). Transcranial focused ultrasound modulates the activity of primary somatosensory cortex in humans. Nat. Neurosci. 17, 322–329. 10.1038/nn.3620 24413698

[B13] Lin W. T. Chen R. C. Lu W. W. Liu S. H. Yang F. Y. (2015). Protective effects of low-intensity pulsed ultrasound on aluminum-induced cerebral damage in Alzheimer's disease rat model. Sci. Rep. 5, 1–7. 10.1038/srep09671 25873429 PMC4397698

[B14] Malayeri A. A. El Khouli R. H. Zaheer A. Jacobs M. A. Corona-Villalobos C. P. Kamel I. R.. (2011). Principles and applications of diffusion-weighted imaging in cancer detection, staging, and treatment follow-up. Radiographics 31, 1773–1791. 10.1148/rg.316115515 21997994 PMC8996338

[B15] Min B. K. Bystritsky A. Jung K. I. Fischer K. Zhang Y. Maeng L. S.. (2011). Focused ultrasound-mediated suppression of chemically-induced acute epileptic EEG activity. BMC Neurosci. 12:23. 10.1186/1471-2202-12-23 21375781 PMC3061951

[B16] Naor O. Krupa S. Shoham S. (2016). Ultrasonic neuromodulation. J. Neural Eng. 13:031003. 10.1088/1741-2560/13/3/031003 27153566

[B17] Pienaar R. Paldino M. J. Madan N. Krishnamoorthy K. S. Alsop D. C. Dehaes M.. (2012). A quantitative method for correlating observations of decreased apparent diffusion coefficient with elevated cerebral blood perfusion in newborns presenting cerebral ischemic insults. Neuroimage 63, 1510–1518. 10.1016/j.neuroimage.2012.07.062 22892333

[B18] Plaksin M. Shoham S. Kimmel E. (2014). Intramembrane cavitation as a predictive bio-piezoelectric mechanism for ultrasonic brain stimulation. Phys. Rev. X 4:011004 10.1103/PhysRevX.4.011004

[B19] Schaefer P. W. Grant P. E. Gonzalez R. G. (2000). Diffusion-weighted MR imaging of the brain. Radiology 217, 331–345. 10.1148/radiology.217.2.r00nv24331 11058626

[B20] Shi C. Y. Lei Y. M. Han H. B. Zuo L. Yan J. H. He Q. Y.. (2015). Transportation in the interstitial space of the brain can be regulated by neuronal excitation. Sci. Rep. 5, 1–11. 10.1038/srep17673 26631412 PMC4668547

[B21] Tufail Y. Matyushov A. Baldwin N. Tauchmann M. L. Georges J. Yoshihiro A.. (2010). Transcranial pulsed ultrasound stimulates intact brain circuits. Neuron 66, 681–694. 10.1016/j.neuron.2010.05.008 20547127

[B22] Tufail Y. Yoshihiro A. Pati S. Li M. M. Tyler W. J. (2011). Ultrasonic neuromodulation by brain stimulation with transcranial ultrasound. Nat. Protoc. 6, 1453–1147. 10.1038/nprot.2011.371 21886108

[B23] Yoo S. S. Bystritsky A. Lee J. H. Zhang Y. Fischer K. Min B. K.. (2011). Focused ultrasound modulates region-specific brain activity. Neuroimage 56, 1267–1275. 10.1016/j.neuroimage.2011.02.058 21354315 PMC3342684

[B24] Yuan Y. Yan J. Ma Z. Li X. (2016a). Effect of noninvasive focused ultrasound stimulation on gamma oscillations in rat hippocampus. Neuroreport 27, 508–515. 10.1097/WNR.0000000000000572 27007778

[B25] Yuan Y. Yan J. Q. Ma Z. T. Li X. L. (2016b). Noninvasive focused ultrasound stimulation can modulate phase-amplitude coupling between neuronal oscillations in the rat hippocampus. Front. Neurosci. 10:348. 10.3389/fnins.2016.00348 27499733 PMC4956652

