# Supplementary material for: Reduced Apparent Diffusion Coefficient in Various Brain Areas following Low-Intensity Transcranial Ultrasound Stimulation
Source: Front Neurosci. 2017 Oct 9;11:562. doi: 10.3389/fnins.2017.00562 (PMC5640877; doi:10.3389/fnins.2017.00562)
Supplement: Supplementary file 1 [file DataSheet1.docx]

Supplementary Material

Reduced apparent diffusion coefficient in various brain areas following low-intensity transcranial ultrasound stimulation

**Yi Yuan ^1†^, Yanchao Dong^2†^, Shuo Hu^1^,Tao Zheng^2^, Dan Du^2^, Juan Du^2^ and Lanxiang Liu^2*^**

*** Correspondence:** Corresponding Author: [liulanxiang66@sina.com](mailto:liulanxiang66@sina.com)

# Supplementary Figures and Tables

For more information on Supplementary Material and for details on the different file types accepted, please see [here](http://home.frontiersin.org/about/author-guidelines#SupplementaryMaterial).

## Supplementary Figure


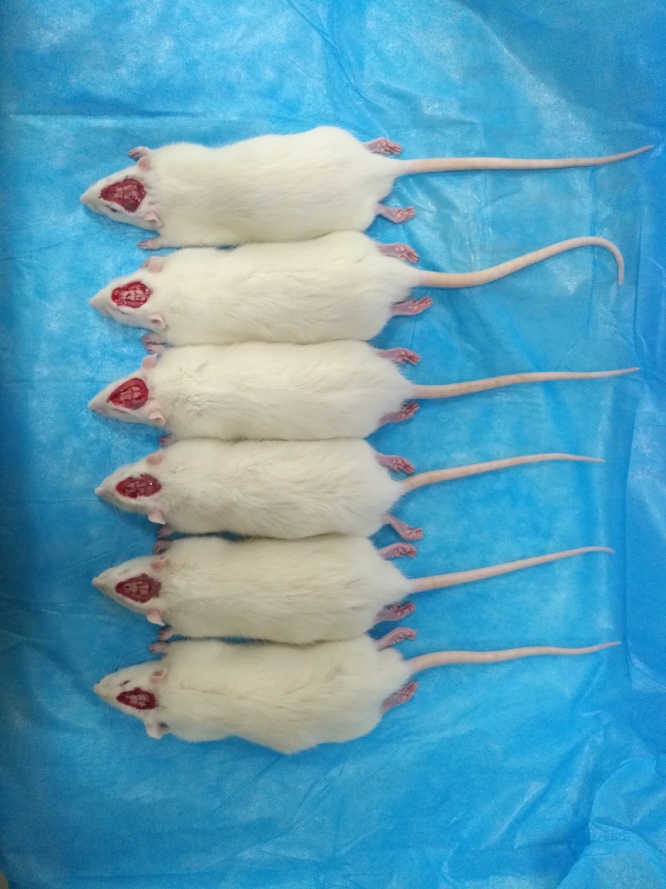


**Supplementary Figure 1.** The photography of the surgery rats

## Supplementary Table

Table 1 (Sham surgery group) The mean ADC values pre- and post-surgery in different ROIs

|  | Mean ADC±S.D (mm^2^ / s) (n=6) | |  |
| --- | --- | --- | --- |
| ROI | pre-surgery | post-surgery | *P* value |
| 1 | 0.752±0.032 | 0.735±0.040 | 0.495 |
| 2 | 0.768±0.043 | 0.758±0.042 | 0.575 |
| 3 | 0.750±0.051 | 0.751±0.034 | 0.961 |
| 4 | 0.743±0.044 | 0.748±0.043 | 0.717 |
| 5 | 0.758±0.034 | 0.748±0.042 | 0.414 |
| 6 | 0.746±0.019 | 0.743±0.040 | 0.851 |
| 7 | 0.705±0.048 | 0.741±0.038 | 0.243 |
| 8 | 0.717±0.061 | 0.749±0.024 | 0.492 |
| ROI 1,2,7,8: CPu | ROI 3,4,5,6: MBmot |  | Friedman test |
